# Immunophenotypic and Gene Expression Analyses of the Inflammatory Microenvironment in High-Grade Oral Epithelial Dysplasia and Oral Lichen Planus

**DOI:** 10.1007/s12105-024-01624-7

**Published:** 2024-03-08

**Authors:** Andres Flores-Hidalgo, James Phero, Scott Steward-Tharp, Megumi Williamson, David Paquette, Deepak Krishnan, Ricardo Padilla

**Affiliations:** 1 Department of Surgery, University of Cincinnati College of Medicine, Cincinnati, OH USA; 2 Department of Pathology and Laboratory Medicine, Emory University School of Medicine, Atlanta, USA; 3 Department of Surgical Sciences, East Carolina University School of Dental Medicine, Greenville, USA; 4 Department of Diagnostic Sciences, University of North Carolina at Chapel Hill Adams School of Dentistry, Chapel Hill, USA

**Keywords:** Oral epithelial dysplasia, Oral lichen planus, Inflammation, Microenvironment

## Abstract

**Background:**

Oral lichen planus (OLP) and oral epithelial dysplasia (OED) present diagnostic challenges due to clinical and histologic overlap. This study explores the immune microenvironment in OED, hypothesizing that immune signatures could aid in diagnostic differentiation and predict malignant transformation.

**Methods:**

Tissue samples from OED and OLP cases were analyzed using immunofluorescence/immunohistochemistry (IF/IHC) for CD4, CD8, CD163/STAT1, and PD-1/PDL-1 expression. RNA-sequencing was performed on the samples, and data was subjected to CIBERSORTx analysis for immune cell composition. Gene Ontology analysis on the immune differentially expressed genes was also conducted.

**Results:**

In OED, CD8 + T-cells infiltrated dysplastic epithelium, correlating with dysplasia severity. CD4 + lymphocytes increased in the basal layer. STAT1/CD163 + macrophages correlated with CD4 + intraepithelial distribution. PD-1/PDL-1 expression varied. IF/IHC analysis revealed differential immune cell composition between OED and OLP. RNA-sequencing identified upregulated genes associated with cytotoxic response and immunosurveillance in OED. Downregulated genes were linked to signaling, immune cell recruitment, and tumor suppression.

**Conclusions:**

The immune microenvironment distinguishes OED and OLP, suggesting diagnostic potential. Upregulated genes indicate cytotoxic immune response in OED. Downregulation of *TRADD*, *CX3CL1*, and *ILI24* implies dysregulation in *TNFR1* signaling, immune recruitment, and tumor suppression. This study contributes to the foundation for understanding immune interactions in OED and OLP, offering insights into future objective diagnostic avenues.

## Introduction

Oral lichen planus (OLP) is a chronic inflammatory condition frequently affecting the oral cavity. Although multiple agents have been previously associated with OLP, no specific etiology is currently described as the cause. The persistent issue in the diagnostic separation between OLP and oral epithelial dysplasia (OED) is the considerable clinical and histologic overlap between the two entities in the oral cavity [1]. There are still no widely accepted diagnostic criteria for OLP. Microscopic differentiation is based upon subjective interpretation of the microscopic presentation by the examining pathologist and the clinical presentation. Although direct immunofluorescence (DIF) studies can help diagnose OLP, DIF has to be correlated with the appropriate clinical presentation and permanent slides, as basement membrane fibrinogen deposition is not pathognomonic of OLP and has been described in OED specimens and other mimickers [2, 3]. Nevertheless, distinguishing between OED with chronic interface mucositis and OLP with reactive cellular atypia can be challenging, necessitating a subjective evaluation of the present morphologic and cytologic features [4].

Oral squamous cell carcinoma (OSCC) is believed to be preceded by a precancerous lesion, mainly defined by clinical visual and histopathologic examination. Based on the presence of OED, these lesions have been grouped under the umbrella term “Oral Potentially Malignant Disorders” (OPMDs). These represent a heterogeneous group of clinical mucosal lesions that may have an increased likelihood of transforming into OSCC [5]. On microscopic examination, the presence and severity of epithelial dysplasia within such lesions are graded from low to high depending on the extent of intraepithelial abnormalities and carry a reported overall risk of malignant transformation of up to 36% [6–8]. Although the presence of dysplasia in biopsied material is a reliable predictor of transformation into OSCC, multiple studies have reported between 0 and 28% of malignant transformations arising from mucosal lesions that do not previously show OED during microscopic examination [7, 9–12].

Other studies have previously shown a direct link between inflammation and malignant transformation [13–15]. The progressive and increasing inflammatory infiltrate density in the microenvironment, alongside qualitative changes in distinct cell populations in OPMD and OSCC, suggests that reversing the environment in OPMD could unveil anti-lesion immunity, leading to immune regression before the onset of OSCC [13, 16].

Evidence indicates that the initial phases of carcinogenesis are linked to alterations in the immune response within the microenvironment [14, 17]. We previously reported a direct link between the presence and distribution of CD4 + and CD8 + lymphocytes and high-grade OED [18]. However, tumor-associated macrophages (TAM) have also been reported to play a role in early carcinogenesis, tumor progression, and later metastasis [19].

Several recent studies have shown increased PD-1/PD-L1 cells in OSCC and OPMD, particularly lesions that progressed to oral cancer [15, 20]. However, accurate interpretation of the immunohistochemical stains can be challenging for the practicing pathologist [21, 22].

Our study hypothesizes that OPMD-induced inflammation can be a surrogate marker to diagnostically differentiate between OED and OLP and to predict malignant transformation. Utilizing immunofluorescence/immunohistochemistry (IF/IHC), we analyzed the presence and distribution of CD4 + and CD8 + infiltrating lymphocytes, CD163/STAT1 + macrophages, and expression of PD-1/PDL-1 in the epithelial compartment of biopsy samples previously diagnosed with OLP and OED. We also performed bulk-RNA sequencing on all the samples and compared the immune genetic expression between OED and OLP.

## Material and Methods

The Institutional Review Boards of the University of North Carolina at Chapel Hill (UNC-CH) and East Carolina University (ECU) registered, reviewed, and approved the study protocol, with the numbers 16-5988 and UMCIRB-21-001256, respectively. All study procedures followed the Declaration of Helsinki and Research Committee Regulations. Oral tissue samples received between July 1, 2005, and January 31, 2022, at the UNC-CH and ECU Oral & Maxillofacial Pathology biopsy tissue archives that were diagnosed by board certified Oral and Maxillofacial Pathologists with oral lichen planus (OLP) and moderate to severe (high-grade) epithelial dysplasia (OED), and met the inclusion criteria, were collected [23]. Biopsy accession files, dental and medical records, and available sample follow-up information were reviewed and collected for each sample. For our study, 10 cases of OED and 10 cases of OLP were identified and collected. The specimens included cases with sufficient tissue for additional analysis from the tongue, buccal mucosa, or floor of the mouth. Tissue samples from patients with a previous history of cancer, prior treatment with steroids and/or immunosuppressed status, subjects under 21 years of age, and patients with a prior history of radiation to the head and neck were excluded. The hematoxylin and eosin (H&E) slides were retrieved and reviewed, and the diagnosis was confirmed by multiple observer consensus. All cases selected for the OLP group were diagnosed according to the diagnostic guidelines published by Cheng et al., currently the American Academy of Oral and Maxillofacial Pathology position paper regarding the diagnosis of OLP, which include patients with intraoral widespread and symmetrical oral lesions, clinical correlation with medication, allergy and chronic use of cinnamon-containing products was ruled out, and incisional biopsies were performed with consistent microscopic and direct immunofluorescence findings [24]. The follow-up information was recorded with the available surgical pathology records with microscopic diagnoses in the same location and respective diagnosis (e.g., invasive carcinoma) or clinical information seen in patient records.

### Immunofluorescence-Immunohistochemistry

Sequential dual immunofluorescence (IF) was performed on paraffin-embedded tissues mounted onto slides. Tissue sections were labeled for the following antigens: dual CD4/CD8 (NCL-L-CD4-368 and NCL-L-CD8-4B11, Leica Microsystems Inc.), STAT1 (9175, Cell Signaling Technology), CD163 (163 M-18, Cell Marque), PDL-1 (13,684, Cell Signaling Technology), and PD-1 (315 M-96, Cell Marque). This assay was carried out on the Leica Bond Rx fully automated slide staining system (Leica Biosystems) using the Bond Research Detection kit (DS9455). Slides were dewaxed in Bond Dewax solution (AR9222) and hydrated in Bond Wash solution (AR9590). Heat-induced antigen retrieval was performed at 100ºC in Bond-Epitope Retrieval solution 1 pH-6.0 (AR9961) for 20 or 10 min. After pretreatment, tissues were blocked, and primary antibodies were diluted. Ready-to-use secondary antibodies, Leica’s Novolink Post Primary and/or Novolink Polymer (RE7260-CE) were used, followed by either TSA Cy5 (SAT705A001EA, Akoya Biosciences) or TSA Cy3 (SAT704A001EA, Akoya Biosciences) to visualize the target of interest. Nuclei were stained with Hoechst 33,258 (Invitrogen). The stained slides were mounted with ProLong Gold antifade reagent (P36930, Life Technologies). Positive controls were included for each assay.

### Histomorphometric Analysis of IF/IHC Slides

All the slides were then scanned in the Aperio ScanScope FL (Leica Biosystems) using a 20X objective. The images were archived in our online database. For all slides, the epithelial region was annotated based on the corresponding overlapping H&E slide. Automated digital analysis of images was run separately in this region. Tissue Studio software (Tissue Studio version 4.2.2 Definiens Inc., Carlsbad CA), specifically the Nuclei and Simulated Cells algorithm in the IF Portal, was used to detect cells that co-expressed biomarkers of interest in the annotated region. The nuclei were digitally detected by Hoechst stain (nuclear counterstain). A cell simulation was performed from these nuclei—cell margins were grown from nuclear boundaries. For this data set, positivity thresholds for the selected antibodies were determined by measuring the average staining intensities inside and outside simulated cells. Measurements were made from six regions of an algorithm training set [18]. The training set images were chosen to encompass the full range of staining intensities in the entire analysis data set (images with high, medium, low, or negative staining). Once thresholds were set, the algorithm evaluated each cell individually for the presence of CD4, CD8, STAT1/CD163, PD-1, and PDL-1.

### RNA-Sequencing

RNA was extracted from FFPE blocks using the truXTRAC FFPE total NA Ultra kit (Covaris). RNA quality with DV200 was examined using the 4200 TapeStation (Agilent Technologies), and RNA concentration was determined using the Qubit Fluorometric Quantitation (Thermo Fisher). Ribosome RNA was depleted using the QIAseq FastSelect (Qiagen), and stranded cDNA library for RNAseq was prepared using the TruSeq Stranded LT mRNA kit (Illumina), following the manufacturer’s protocols. Pooled libraries were paired-end sequenced (100 bp × 2) on the NextSeq 2000 system (Illumina). Raw sequence reads were de-multiplexed by the on-instrument DRAGEN (v3.8.4). Sequence reads of each sample were pseudo-aligned to the human hg38 reference transcriptome, and the gene transcript abundance was quantified using the Kallisto (v0.48.0). Differential gene expression was achieved using biomaRt (v2.50.3), tximport (v1.22.0) and DESeq2 (v1.34.0) packages in the R Studio (Build 386 with R v4.1.1).

### Data Analysis

Morpheus (Board Institute, Cambridge BA), a web-based visualization software, was used for hierarchical clustering analysis of the differentially expressed genes. Gene ontology and enrichment analysis were performed to explore the function and product of differential gene expressions in both study groups. The *p*-value of < 0.05 and fold change > 1 was set for this purpose utilizing the Gene Ontology knowledgebase by the Gene Ontology Consortium (release date 2023-01-05) [25–27].

## Results

### Patient Cohort

A total of 20 samples that met our inclusion criteria were selected for the study, 10 cases of each group (OED and OLP, respectively). Patients’ demographics and clinical and histopathologic diagnoses in both study groups are presented in Table 1.

**Table 1:** Study subjects’ demographics and corresponding microscopic diagnosis of each sample

Case	Age	Sex	Clinical presentation	Microscopic diagnosis	Biopsy site	Follow-up
1	50	F	Erythroleukoplakia	Moderate epithelial dysplasia	Buccal mucosa	Not available
2	72	M	Erythroleukoplakia	Severe epithelial dysplasia	Buccal mucosa	6 m—recurrence
3	56	M	Leukoplakia	Moderate epithelial dysplasia	Ventral tongue	Not available
4	69	M	Leukoplakia	Moderate epithelial dysplasia	Buccal mucosa	Not available
5	61	F	Leukoplakia	Moderate epithelial dysplasia	Buccal mucosa	11 months—no recurrence
6	73	M	Leukoplakia	Moderate epithelial dysplasia	Buccal mucosa	Not available
7	56	M	Leukoplakia	Severe epithelial dysplasia	Lateral tongue	16 m—minimally invasive OSCC
8	80	M	Erythroleukoplakia	Moderate epithelial dysplasia	Lateral tongue	6 m—no recurrence
9	71	M	Leukoplakia	Moderate epithelial dysplasia	Ventral tongue	7 m—no recurrence
10	68	M	Leukoplakia	Moderate epithelial dysplasia	Buccal mucosa	33 m—OSCC recurrence
11	71	M	Bilateral tongue leukoplakia	Oral lichen planus	Lateral tongue	Not available
12	68	F	Leukoplakia	Oral lichen planus	Buccal mucosa	15 m—under treatment
13	39	M	Erythroleukoplakia	Oral lichen planus	Buccal mucosa	8 m—under treatment
14	73	F	Multifocal leukoplakia	Oral lichen planus	Junction of vestibule and gingiva	Not available
15	68	M	Leukoplakia	Oral lichen planus	Floor of mouth	6 m—under treatment
16	57	M	Erythroleukoplakia	Oral lichen planus	Buccal mucosa	Not available
17	50	F	Leukoplakia	Oral lichen planus	Buccal mucosa	23 m—patient developed sjogren syndrome
18	80	M	Leukoplakia	Oral lichen planus	Upper lip	Not available
19	62	F	Erythroleukoplakia	Oral lichen planus	Lateral tongue	Not available
20	71	F	Leukoplakia	Oral lichen planus	Buccal mucosa	Not available

### Presence of Intraepithelial CD4 and CD8 Cells in Oral Lichen Planus versus High-Grade Dysplasia

**Fig. 1:**
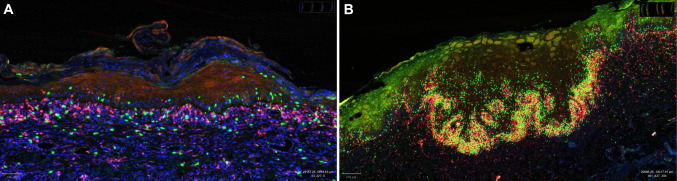
Differences in the infiltration pattern of CD4 and CD8 + lymphocytes in oral lichen planus (**A**) and epithelial dysplasia (**B**). Direct immunofluorescence—CD4 Cy5 channel (red), CD8 Cy3 channel (green) and DAPI nuclear (blue)]

We observed results similar to our previous study. In the OED group, the most notable infiltration of CD8 + T-cells was evident, with these cells observed to infiltrate the dysplastic epithelium and track the atypical changes. Since all samples in this group exhibited moderate to severe dysplasia, CD8 + cells were specifically localized in the region spanning the basal layer to the upper half and upper third of the epithelial thickness. The patterns of infiltration of CD8 + also corresponded with the level of dysplasia within the epithelium. There was a moderate increase in the inflammatory cell infiltrate in the OED group compared to oral OLP. CD8 + cell distribution was strikingly different between OLP and high-grade OED. There is also a mild increase in the presence of CD4 + lymphocytes in the basal layer of the epithelial compartment in the OED samples (Fig. 1).

### Intraepithelial CD4/CD8 Ratio in OED vs OLP

Software-based histomorphometric analysis of the immune cells highlighted by IF/IHC revealed an average of 1884.2 CD4 + infiltrating cells in all OEDs, accounting for 34.2% of all immune cells. The average of CD8 + intraepithelial lymphocytes was 3624.4 reactive cells, representing 65.8%. In the intraepithelial compartment of OLP cases, we encountered an average of 1391.5 CD4 + and 1902 CD8 + cells, representing 42.2% and 57.8%, respectively. This constitutes a CD4/CD8 ratio of 1:2 in OED and approximately a 1:2 ratio of intraepithelial CD4/CD8 immune cells in OLP.

### The Presence of Infiltrative Tumor-Associated Macrophages in OED and OLP

**Fig. 2:**
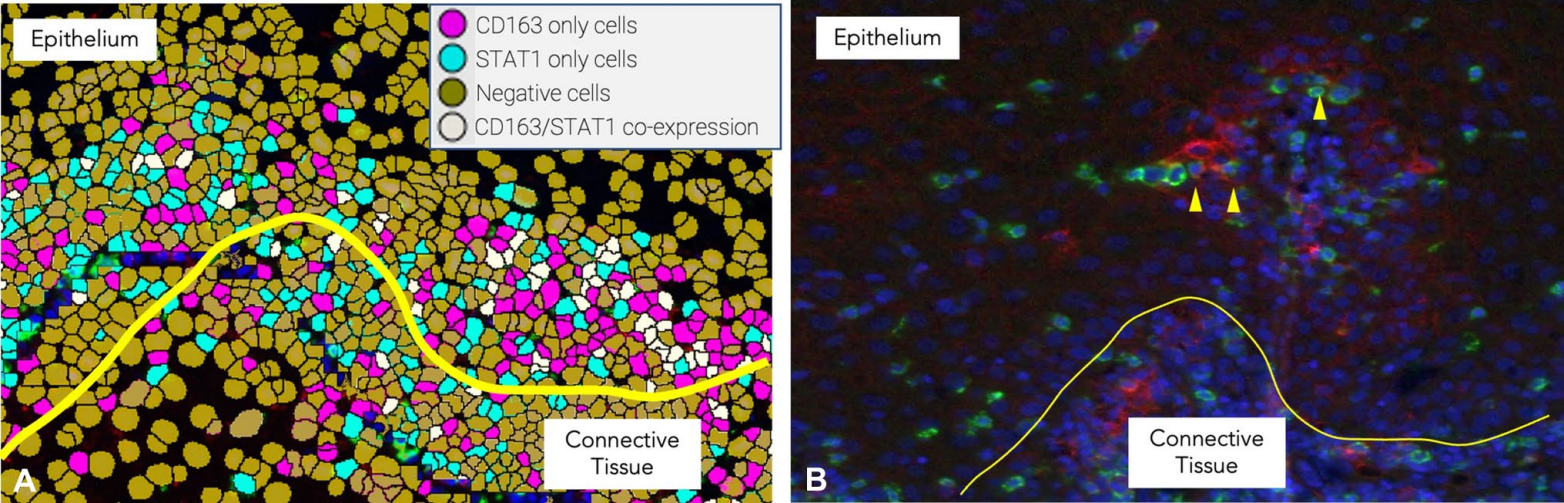
**A** Software-driven histomorphometric analysis in utilized to identify CD163/STAT1 + macrophages within the epithelial compartment. **B** DIF counterpart. The yellow arrowheads point at double-positive macrophages with overlapping stains [Direct immunofluorescence (yellow line highlights the basements membrane)—CD163 Cy5 channel (red), STAT1 Cy3 channel (green), and DAPI nuclear stain (blue). Yellow lines highlights the basement membrane]

To further characterize the phenotype of the inflammatory cell population surrounding OLP and OED, we examined the coexpression of TAM using double-labeling IF/IHC for CD163/STAT1 in both groups. In OED, macrophages expressing STAT1/CD163 + were predominantly located beneath the epithelium and showed a mild correlation with the distribution of CD4 + cells in the same tissue layer. STAT1/CD163 + macrophages were mainly distributed in the subepithelial region in the OLP group. The number of intraepithelial double-positive TAM was also slightly increased in moderate to severe dysplasia and correlated with similar patterns of infiltration of CD8 + lymphocytes and the level of dysplasia progression in the covering tissue (Fig. 2).

The average of double-positive STAT1/CD163 + cells in OED was 148 of all reactive cells, which accounted for 55.1%. Fewer of these activated macrophages were encountered in the OLP group, with an average of 98.2, which accounts for 34.3% of all immunoreactive cells in the intraepithelial compartment.

### Expression of PD-1/PDL-1 Markers in OED versus OLP

**Fig. 3:**
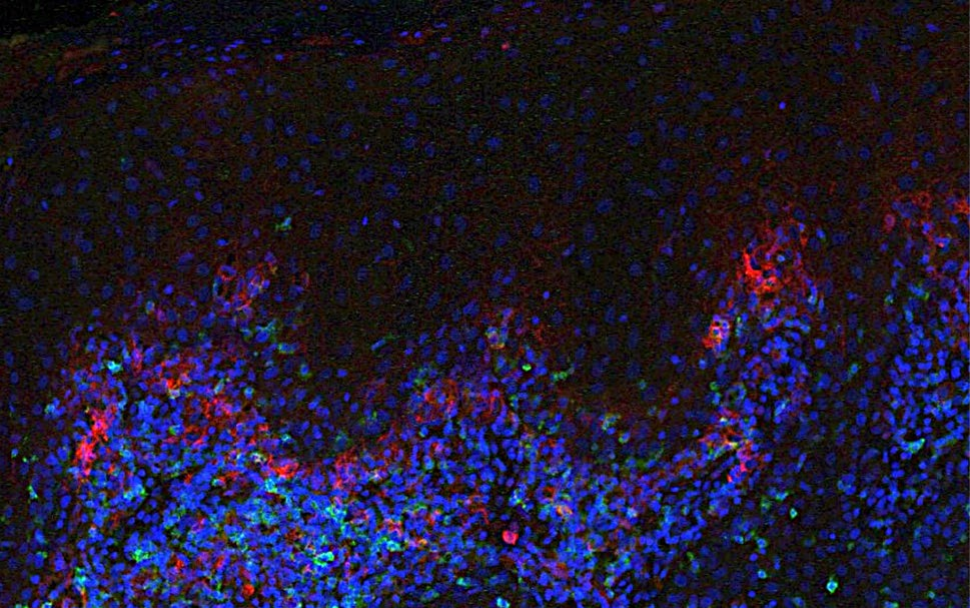
Expression of PD1 and PDL1 in a high-grade OED sample. [Direct immunofluorescence—PDL1 Cy5 channel (red), PD1 Cy3 channel (green) and DAPI nuclear stain (blue)]

No general differences between PD1/PDL1 expression were found in OLP versus OED. Software histomorphometric analysis found that PD1 was expressed on CD4 + and CD8 + T cells. However, PDL1 expression increased in a subset of OED compared to OLP samples (Fig. 3). When looking at the follow-up information available, this finding does not correlate with progression to OSCC. Also, a correlation between PD1 expression and CD4 + cells was identified in 8 out of the 10 OED selected cases.

### RNA-Sequencing

#### Total Lymphoid Composition in OED and OLP

**Fig. 4:**
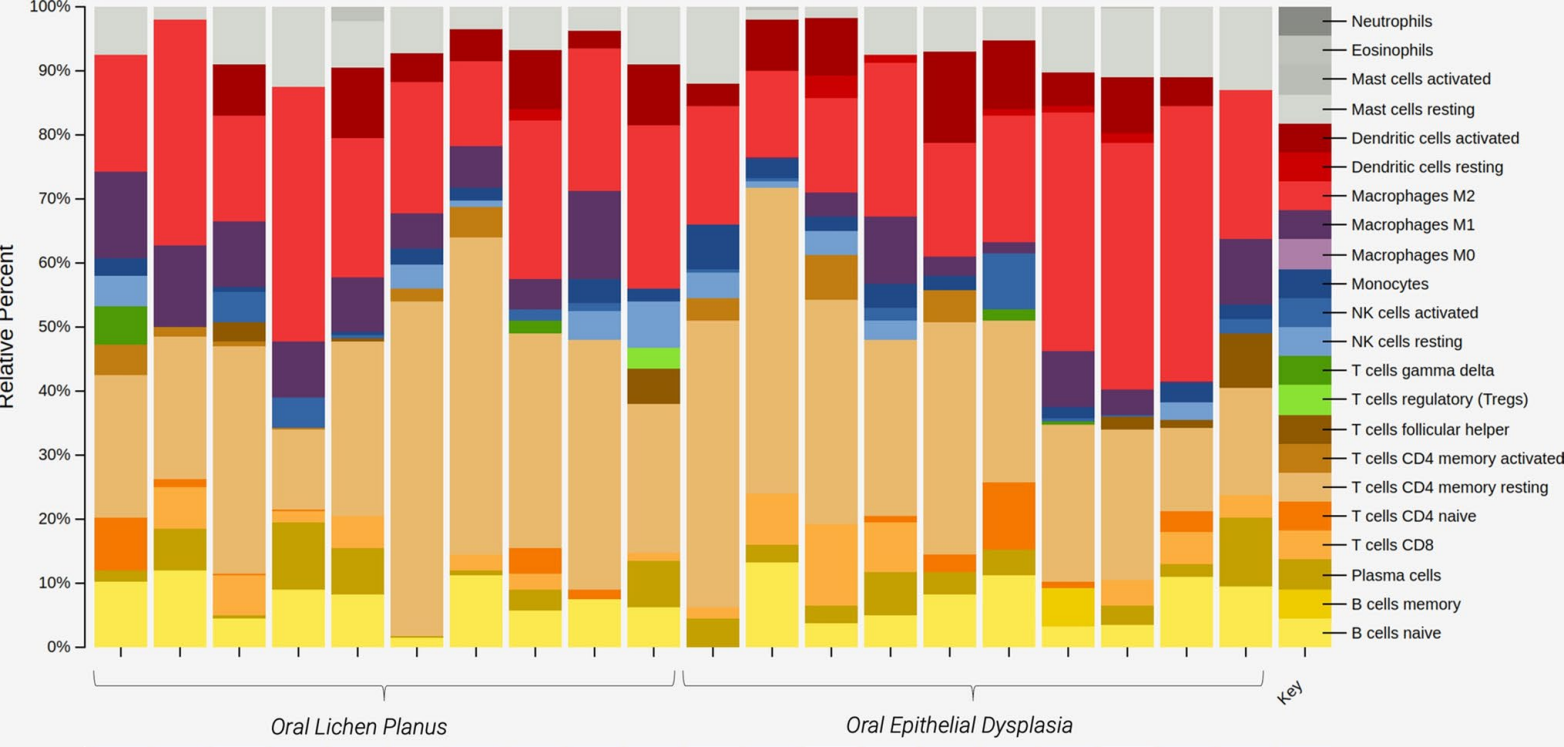
Relative percentages of inflammatory cells present, identified by bulk RNA-sequencing. Comparison between oral lichen planus group (left) and oral epithelial dysplasia (right)

To determine the cellular composition in each sample, we utilized CIBERSORTx analysis on the RNA sequencing data [28]. The relative proportions of 22 immune cell types were deciphered using the predefined LM22 signature gene matrix, with parameters set at 1000 permutations and B-batch correction. The overall lymphoid composition was calculated by assessing the relative percentages of various cell types, including neutrophils, eosinophils, activated and resting mast cells, activated and resting dendritic cells, M1 and M2 macrophages, monocytes, activated and resting natural killer cells, gamma delta T-cells, regulatory T-cells (Tregs), T-cell follicular helper cells, activated and resting CD4 memory cells, CD8 cells, plasma cells, B-cell memory cells, and native B-cells. The visual representation of these percentages is depicted in Fig. 4. No statistically significant difference was observed in the total lymphoid composition between the selected cases of OED and OLP (*p* > 0.05).

**Fig. 5:**
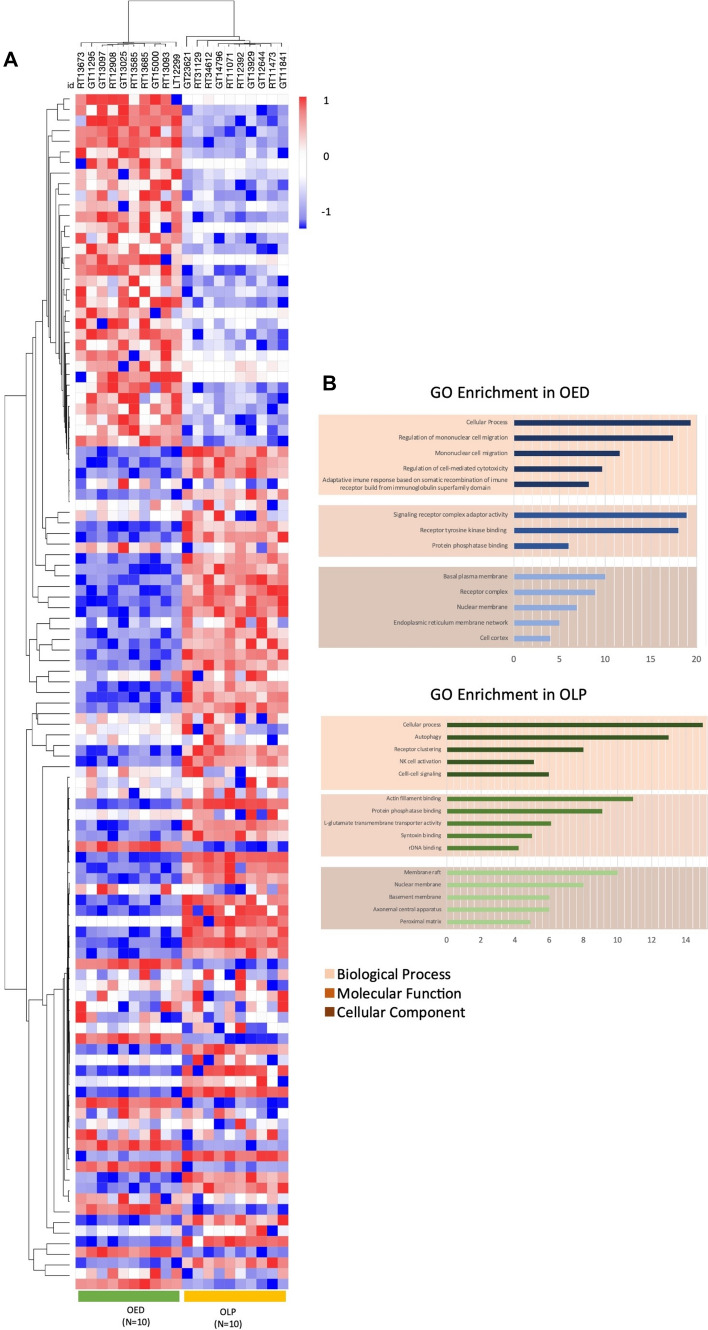
**a** Cluster heatmap of the 113 differentially expressed genes in each sample of OED and OLP. **b** Gene ontology enrichment analysis of the differentially expressed genes in both study groups

Since immune-related genes have been reported to be upregulated in high-grade OED [29–32], we compared the immune phenotype and function to previously described cancer-associated immune signatures*.* We also focused on analyzing immune signatures between both groups and the previously reported genetic expression of tumor-infiltrating leukocytes [29, 33]. According to the sequencing data, we obtained 81 differentially expressed genes, of which 33 were upregulated, and 48 were downregulated in moderate-severe OED. We found 31 differential genes in the OLP group, of which 11 were upregulated and 20 were downregulated. A cluster heat map and volcano plot of the differentially expressed genes in each study group can be seen in Fig. 5a and b.

#### Gene Ontology Analysis

Functional analysis revealed enrichment of immune signatures associated with immunosurveillance, lymphocyte infiltration, cytotoxic response, and surrogate markers of tumor-associated macrophages in high-grade OED. In OLP, our data revealed differential gene expression mainly related to lymphocyte infiltration, T-cell regulation, and cytotoxicity. The results of the biological process, molecular function, and cellular component analysis for both groups are visually laid out in Fig. 5.

## Discussion

OPMDs encompass a heterogeneous group of lesions and conditions that exhibit an increased risk of transforming into oral cancer. The global incidence of OPMDs varies across populations, with factors such as age, gender, tobacco and alcohol consumption, and socioeconomic status contributing to regional disparities. Studies have consistently shown a higher prevalence of OPMDs in older individuals, especially those engaging in tobacco and alcohol use [34]. Various disorders may exhibit clinical and histopathological traits akin to OED and OLP [35, 36]. While OED typically manifests as a solitary lesion with varying proportions of white and red color changes and ulceration, a multifocal presentation is well-recognized, particularly in patients with advanced tobacco-related mucosal injury and proliferative verrucous leukoplakia (PVL) [37].

A band-like chronic inflammatory cell infiltrate in the superficial lamina propria commonly accompanies OED. When viewed under low-magnification microscopy, this can closely resemble the histopathological features of OLP [38]. Substantial clinical and histologic overlap has been observed between OLP and OED of the oral cavity, particularly in cases of PVL. This raises questions about whether purported transformations of oral lichen planus to squamous cell carcinoma represent undetected premalignant lesions with a nonspecific inflammatory response mimicking OLP [9, 39].

The infiltration of various immune cells, including lymphocytes, macrophages, and dendritic cells, mark the immune microenvironment of OED. These cells play a pivotal role in the surveillance and response to dysplastic changes. Studies have demonstrated an increased density of CD8 + cytotoxic T cells in OED lesions, suggesting their involvement in antitumor immunity [40]. Additionally, regulatory Tregs have been noted, indicating a complex balance between effector and suppressor immune responses [16]. The immune microenvironment of OED reflects a dynamic struggle between immunosurveillance and immune evasion mechanisms. While an influx of immune cells indicates an attempt to eliminate dysplastic cells, OED lesions often employ immune evasion strategies, including upregulation of programmed death-ligand 1 (PD-L1) on dysplastic epithelial cells, dampening the antitumor immune response [41].

Very few studies have focused on identifying molecular alterations in the immune microenvironment surrounding OPMD. The abundance and composition of intraepithelial and underlying immune cells in OPMD may play a role in predicting cancer progression, prognosis, and possible immunotherapy as a preventive measure. Hence, the genomic and molecular profile of said immune intraepithelial cells is still needed. Regarding the epithelial compartment, cytogenetic alterations such as copy number gain of 16q, 8q, and loss of 3p, 8p, 9p, 4q, 5q, and 13q are biomarkers for premalignant oral lesions [42]. Also, protein overexpression and mutation of the *p53* gene have been explored in OPMD [43, 44]. However, these molecular alterations have yet to be translated into clinical utility because their effectiveness as a biomarker is limited [45, 46], as chemoprevention may prove ineffective when the target cell population carries a significantly high risk or has already undergone preneoplastic irreversible cellular changes [46, 47]. Hence, exploring the relationship between epithelial dysplastic changes and the immune response profile in the microenvironment setting for planning a targeted treatment can be useful for diagnostic and, later, management purposes.

Our study only analyzed immune signatures in both study groups. Gene Ontology studies provide a systematic and standardized framework for annotating and categorizing genes, facilitating a deeper understanding of gene function, biological processes, and their implications in health and disease. According to our GO analysis, aberrant expression in OED was mainly associated with immune signatures associated with immunosurveillance, lymphocyte infiltration, cytotoxic response, and surrogate markers of tumor-associated macrophages. These results are in accordance with the existing bulk RNA-sequencing studies that have facilitated the identification of specific immune cell populations infiltrating OED lesions. Notably, increased expression of genes associated with cytotoxic T lymphocytes, such as *CD8A* and *granzyme B* (*GZMB*), has been observed in previous studies [41, 48].

The top upregulated genes were *NKG7*, *MS4A4A*, *CD8A*, *MST1R*, and *NCR3LG1*. *CD8A* encodes the CD8 alpha chain, a cell surface glycoprotein expressed on cytotoxic T cells. It plays a key role in antigen recognition and the immune response against infected or abnormal cells [49]. *MST1R* encodes the receptor for macrophage-stimulating protein, and it is involved in various cellular processes, including cell survival, migration, and immune responses [50]. Similar findings have been reported upregulated in similar studies in high-grade dysplasia or early OSCC [48, 51, 52]. These findings were also corroborated by the increased intraepithelial infiltration of CD8 + lymphocytes and CD163/STAT1 + macrophages by IHC/DF in all cases of high-grade OED versus OLP (Fig. 1). This suggests an active involvement of cytotoxic T cells in the immune response against dysplastic cells in OED. Interestingly, the infiltration of said immune cells follows the dysplastic changes within the intraepithelial tissue. STAT is an IFN-inducible product, but specifically, the activation of STAT1 in TAM might lead to the up-regulation of inducible nitric oxide synthase and Arginase I activity. Nitric oxide synthase and Arginase could play a role in suppressing T cells. This is why STAT1 was chosen as an antibody for identifying TAM in our study [53, 54].

Upregulation of *NKG7* and *MST1R* also indicates a cytotoxic nature of the immune microenvironment elicited by the premalignant changes occurring in the epithelium. Hence, we can consider that the RNA-sequencing data may have originated from T- and NK-enriched parts rather than all of the OED tissues. This makes sense, as cytotoxic cells will eliminate keratinocytes undergoing malignant changes in the epithelial compartment. Moreover, the coexpression of CD163 and STAT1 in macrophages in OED suggests a pro-inflammatory phenotype; hence, it is likely that infiltrated CD4 + cells modulate the phenotype of associated macrophages in oral premalignant lesions.

It is also important to highlight that in a small portion of the OED cases when taken together, immune signatures reported as being involved in immune suppressive mechanisms, such as Treg cells and PD1/PDL1, were enriched. However, no significant overexpression of PD1/PDL1 was identified by IHC/DIF, nor clinical correlation with malignant transformation, when compared with available follow-up information.

The most common downregulated genes in OED were *TRADD*, *CX3CL1*, and *ILI24*. While there might not be specific studies on the downregulation of these genes in OED, the general understanding of their roles in cancer suggests that their downregulation could contribute to dysplastic changes in oral epithelial cells. Dysregulation of apoptosis and inflammation are key features in the progression of precancerous lesions like OED. The *TNFRSF1A-associated death domain* or *TRADD* gene mediates cellular responses to tumor necrosis factor receptor 1 (TNFR1) signaling. While direct studies on the downregulation of *TRADD* in carcinogenesis may be limited, alterations in TNFR1 signaling pathways, including *TRADD*, have been implicated in various malignant neoplasms. Some studies suggest that dysregulation of TNFR1 signaling, which includes TRADD, can contribute to cancer development and progression [55]. *CX3CL1* also plays a role in the recruitment and activation of immune cells within the tumor microenvironment. Its downregulation can contribute to an immunosuppressive milieu, allowing tumors to evade immune surveillance. Reduced *CX3CL1* expression has been associated with enhanced tumor invasion and metastasis. The interaction between *CX3CL1* and *CX3CR1* regulates the adhesion of immune cells to tumor cells, influencing the metastatic potential of cancer [56, 57]. *Interleukin 24* (*IL24*) has been extensively implicated in carcinogenesis, affecting various aspects of cancer development. Downregulation of *IL24* has been associated with multiple cancer types, and its tumor-suppressive properties are attributed to its ability to induce apoptosis and inhibit tumor cell growth [58].

In contrast for OLP, our data revealed differential gene expression mainly related to lymphocyte infiltration, T-cell regulation, and cytotoxicity. Reported findings consistently reveal gene alterations associated with immune responses, inflammation, and epithelial integrity [59]. Similar studies have also found dysregulation in signaling pathways related to inflammation, immune responses, and cell adhesion [60]. The top upregulated genes in the OLP group were *TCIRG1*, *DCG1*, and *NRIP1*.

All these genes regulate immune responses, particularly in T-cell and dendritic cell function. While direct evidence of upregulation in OLP may not be readily available, the involvement of immune regulatory genes like *TCIRG1* and *DCG1* in their function in immune regulation suggests a possible role in the inflammatory processes associated with OLP. However, specific studies on its upregulation in OLP are limited [60].

Our limitations in this pilot study included the retrospective nature of the project and limited research funds, which prevented the inclusion of more cases in both study groups, as well as low-grade dysplasia and invasive OSCC groups. Another limitation was the lack of follow-up information or longer follow-up time since the initial microscopic diagnosis to assess malignant transformation in many cases. Many of the patients in the OED group were referred to outside institutions for definitive treatment after the incisional biopsy and diagnosis were made; hence, we did not have access to this information.

## Conclusion

The immune microenvironment of high-grade OED is a dynamic and intricate network involving immune cells, cytokines, and inflammatory mediators. Elucidating these interactions is crucial for unraveling OED pathogenesis, predicting malignant transformation, and developing targeted therapeutic strategies. Our preliminary data suggests that the inflammatory microenvironments associated with OED and OLP differ; as such, they could be used for objective and accurate diagnostic separation during microscopic examination in the future. Further research is necessary, including analyzing more cases (comprising hyperkeratosis/reactive mucosal lesions, low-grade OED, and invasive OSCC) and spatial transcriptomics.

## Data Availability

No datasets were generated or analysed during the current study.
